# Perforation of the descending colon diverticulum in a patient following recovery from severe COVID-19 pneumonia: a case report

**DOI:** 10.1093/jscr/rjab013

**Published:** 2021-02-18

**Authors:** Michinori Hamaoka, Shohei Shiozaki, Hideki Nakahara, Toshiyuki Itamoto

**Affiliations:** Department of Gastroenterological Surgery, Hiroshima Prefectural Hospital, Hiroshima, Japan; Department of Gastroenterological Surgery, Hiroshima Prefectural Hospital, Hiroshima, Japan; Department of Gastroenterological Surgery, Hiroshima Prefectural Hospital, Hiroshima, Japan

## Abstract

The outcome of surgery in patients who have recovered from severe coronavirus disease 2019 (COVID-19) is unknown. Herein, we present a case of an emergency operation for acute pan-peritonitis due to perforation of the descending colon diverticulum in a patient who recovered from severe COVID-19 pneumonia. A 59-year-old man, who had recovered from severe COVID-19 pneumonia ~6 months previously, developed acute pan-peritonitis due to perforation of a diverticulum in the descending colon. Emergency surgery was performed, and the perforation was sutured and closed. He was discharged from the hospital 13 days postoperatively. There was no relapse of COVID-19 during the perioperative period of peritonitis surgery. General perioperative management may, therefore, be sufficient in patients who have recovered from COVID-19.

## INTRODUCTION

The health emergency linked to the coronavirus disease 2019 (COVID-19) infection represents an absolutely new problem for all health professionals. In particular, information regarding the spread of the virus and its manifestations in the gastroenterological surgery field is still incomplete. There are few reports of surgery following patient recovery from severe COVID-19 pneumonia. We present a case of an emergency operation for acute pan-peritonitis due to perforation of a descending colon diverticulum in a patient who had recovered from severe COVID-19 pneumonia.

## CASE REPORT

**Figure 1:**
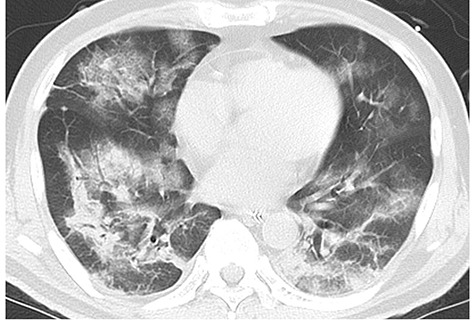
Chest CT at the onset of COVID-19 showing bilateral patchy ground-glass opacities and infiltrative shadows.

**Figure 2:**
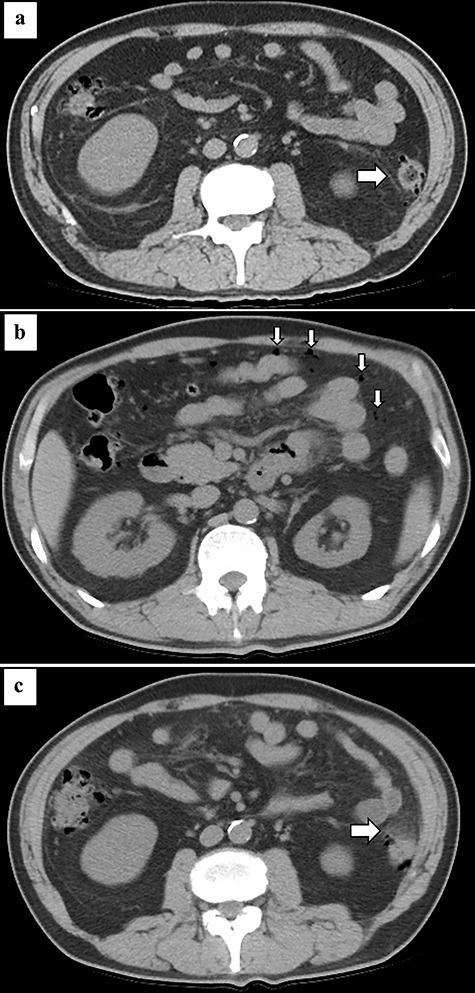
Abdominal CT at the onset of COVID-19 showing diverticulum of the descending colon (arrow) (**a**). Abdominal CT at the onset of pan-peritonitis showing free air in the abdominal cavity (arrow) (**b**) and increased fat density surrounding the descending colon (arrow) (**c**).

**Figure 3:**
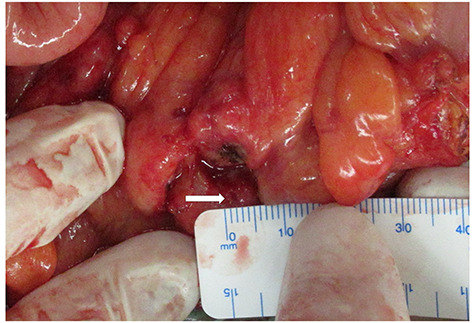
Pinhole perforation in the descending colon is observed (arrow).

A 59-year-old man with a history of hypertension, type II diabetes mellitus, old myocardial infarction and chronic heart failure suffered from COVID-19 pneumonia 8 months prior to admission (Fig. 1). He required an artificial respirator on the ninth day of onset due to worsening of respiratory status. He underwent tracheotomy on the 19th day of onset. Two COVID-19 polymerase chain reaction (PCR) tests conducted on the 32nd day after the initial infection showed negative results. He was discharged on the 52nd day of onset. Six months after discharge, he presented with sudden abdominal pain and was admitted to our hospital. His vital signs were temperature, 35.1°C; heart rate, 74 beats per minute; blood pressure, 121/92 mmHg; and SpO2, 100% on room air. Physical examination showed board-like rigidity and rebound tenderness over the entire abdomen. Laboratory results showed significant increase in inflammatory response, with a white blood cell count of 18 000/μl. Computed tomography (CT) revealed ascites, free air in the abdominal cavity, multiple diverticula of the descending colon (Fig. 2b) and increased fat density surrounding the descending colon (Fig. 2c). We diagnosed the patient with colonic perforation and performed emergency surgery. Surgical findings revealed pinhole perforation in the descending colon (Fig. 3). As the perforation was small and there was no fragility of the surrounding colonic tissue, the perforation was sutured and closed. Then, the sutured site was covered with epiploicea appendices. A drain was placed in the left paracolic gutter after abdominal cleaning. The abdominal wall was closed without a diverting stoma. The patient was wheezing on the first postoperative day, and he required oxygenation when SpO2 dropped to 70% on room air. His COVID-19 PCR test was negative. The cardiothoracic ratio, which was 40% preoperatively, increased to 50%. He was diagnosed with exacerbation of chronic heart failure and was treated with diuretics, and his symptoms improved. He passed without any complications, and he was discharged from the hospital 13 days after the operation.

## DISCUSSION

The environment surrounding medical care has changed significantly due to the COVID-19 pandemic. It has had a significant impact on the surgical field. Patients with perioperative viral infection have a higher rate of mortality, and half of these patients have postoperative pulmonary complications [1]. Therefore, surgeons should be very careful about perioperative infection in patients undergoing surgery. The results of emergency surgery performed on cases long after recovery from severe COVID-19 pneumonia are currently unknown.

Although acute pan-peritonitis due to diverticulum perforation is a common disease, the association between COVID-19 and this disease is unclear. In this case, the descending colonic diverticulum was present at the time of onset of COVID-19 (Fig. 2a), so COVID-19 did not affect diverticulum formation. However, COVID-19 has been shown to cause gastrointestinal symptoms, such as vomiting, diarrhea or abdominal pain during the early phases of the disease. Intestinal dysfunction induces changes in intestinal microbes [2]. These changes can cause diverticulitis. In the present case, diverticulitis developed more than half a year after recovery from COVID-19, so the direct association with COVID-19 might be low.

The present case had deterioration of oxygenation and required oxygen on the first postoperative day. There was a report that patients who had recovered from COVID-19 showed positive PCR test results after discharge from the hospital [3]. The possibility of recurrence was considered in this case as well; however, the PCR test was negative. This patient had a history of old myocardial infarction and chronic heart failure. During recovery from COVID-19, he had no exacerbation of heart failure. Therefore, it is highly possible that the stress of pan-peritonitis, surgery and fluid loading temporarily exacerbated heart failure postoperatively. As there are reports of myocardial damage due to COVID-19 [4], it would be desirable to evaluate cardiac function preoperatively in surgical cases.

There have been reports of COVID-19 and death after surgery for diverticulitis [5]. Perioperative viral infection can lead to serious lung disease, and more intensive care than general postoperative management is required. The present surgical case after recovery from COVID-19 did not require intensive care. While this case suggests that general perioperative management is sufficient in cases following recovery from COVID-19, the collection of future cases is desired.

In conclusion, we performed emergency surgery for acute pan-peritonitis due to perforation of the descending colon diverticulum in a patient with a history of severe COVID-19 pneumonia about half a year ago. There was no recurrence of COVID-19 during the perioperative period, and perioperative management was possible with general measures.

## CONFLICT OF INTEREST STATEMENT

None declared.

## FUNDING

None.

## References

[1] Nepogodiev D, Glasbey JC, Li E, Omar OM, Simoes JFF, Abbott TEF, et al. Mortality and pulmonary complications in patients undergoing surgery with perioperative SARS-CoV-2 infection: an international cohort study. Lancet (London, England) 2020;396:27–38. 10.1016/S0140-6736(20)31182-X PMC7259900 32479829

[2] Villapol S. Gastrointestinal symptoms associated with COVID-19: impact on the gut microbiome. Translational research: the journal of laboratory and clinical medicine 2020;226:57–69. 32827705 10.1016/j.trsl.2020.08.004 PMC7438210

[3] Zhang J, Qu H, Li C, Li Z, Li G, Tian J, et al. More caution needed for patients recovered from COVID-19. Front Public Health 2020;8:562418. 33224913 10.3389/fpubh.2020.562418 PMC7667189

[4] Puntmann VO, Carerj ML, Wieters I, Fahim M, Arendt C, Hoffmann J, et al. Outcomes of cardiovascular magnetic resonance imaging in patients recently recovered from coronavirus disease 2019 (COVID-19). JAMA Cardiol 2020;5:1265–73. 32730619 10.1001/jamacardio.2020.3557 PMC7385689

[5] Montali F, Palmieri G, Casali L, Pagliai L, Costi R. Rapidly fatal outcome of Covid-19 after successful emergency surgery during pandemic outbreak in northern Italy. Int J Surg Case Rep 2020;73:9–12. 32622186 10.1016/j.ijscr.2020.06.073 PMC7305717

